# DEP-TFDualNet: A Dual-Domain Attention Framework with Temporal–Frequency Fusion for Depression Recognition Using Three-Channel Frontal EEG

**DOI:** 10.3390/s26123861

**Published:** 2026-06-17

**Authors:** Haijun Lin, Jiayi Liu, Dongxu Jiang

**Affiliations:** Heilongjiang Province Key Laboratory of Laser Spectroscopy Technology and Application, Harbin University of Science and Technology, Harbin 150080, China; 2420610198@stu.hrbust.edu.cn

**Keywords:** depression recognition, electroencephalography, frontal EEG, resting-state EEG, temporal–frequency fusion, deep learning

## Abstract

**Highlights:**

**What are the main findings?**

- Subject-level depression recognition was feasible under a fixed three-channel frontal resting-state EEG configuration using DEP-TFDualNet.
- In the evaluated cohort, DEP-TFDualNet achieved the best threshold-based subject-level performance and the lowest Brier score among the compared models.

**What are the implications of the main findings?**

- The results provide preliminary evidence that simplified frontal EEG sensing may support depression recognition in acquisition-constrained settings.
- The proposed framework may facilitate future research on portable EEG-assisted depression screening with low-channel sensor configurations.

**Abstract:**

Early depression screening is important for timely intervention, and electroencephalography (EEG) offers an objective and potentially portable sensing modality for computer-aided assessment. However, recognition from fixed three-channel frontal EEG remains difficult because of limited spatial information and incomplete modeling of temporal–frequency characteristics and temporal dependencies. This study proposes DEP-TFDualNet for acquisition-constrained frontal resting-state EEG. The framework integrates multi-scale convolution, dual-domain channel attention, temporal modeling derived from the independent recurrent neural network (IndRNN) architecture, and decision-stage fusion of deep representations with low-order statistical descriptors through a Kolmogorov–Arnold Network (KAN)-based nonlinear projection layer. Experiments were conducted on the publicly available three-channel frontal EEG subset of the MODMA dataset. After additional quality control, 48 subjects were retained (22 patients with major depressive disorder, 26 healthy controls). Under subject-wise stratified five-fold cross-validation, DEP-TFDualNet achieved 85.42% accuracy, 85.26% macro-F1, 81.82% sensitivity, 88.46% specificity, an AUC of 0.82, and a Brier score of 0.121. It achieved the best threshold-based subject-level performance and the lowest Brier score among the evaluated models. These results provide preliminary evidence that simplified frontal EEG sensing may support depression recognition in acquisition-constrained settings, although larger and external validation is still required.

## 1. Introduction

Depression is a common and high-burden mental disorder that substantially impairs psychological well-being and social functioning and has become a major public health concern [1]. Its high prevalence and recurrence further contribute to adverse long-term outcomes [2]. Owing to the heterogeneity of its clinical manifestations, current diagnosis and assessment still rely primarily on clinical interviews and rating scales, which are susceptible to subjectivity and assessment bias [3,4,5]. Therefore, objective and quantifiable physiological biomarkers are of considerable value for computer-aided depression recognition and early screening. Among available modalities, electroencephalography (EEG) has attracted increasing attention because it is noninvasive, low-cost, portable, and capable of capturing brain activity with high temporal resolution [6].

EEG-based depression recognition has progressed from conventional pipelines based on handcrafted features and machine learning toward deep-learning-based representation learning frameworks [7]. Traditional approaches often depend heavily on manually designed features and may have limited ability to capture the nonlinear dynamics of EEG signals. By contrast, recent deep learning approaches, including convolutional, recurrent, hybrid, and attention-based architectures, can learn discriminative representations directly from raw signals or transformed inputs and have shown promising performance in depression recognition tasks [8,9,10].

In addition to model architecture, EEG channel configuration strongly affects both practical deployment and modeling strategy. Multi-channel EEG provides richer spatial information but usually requires more complex hardware, longer preparation time, and higher deployment cost, which limit its use in portable screening scenarios. By contrast, few-channel EEG is more attractive for practical applications because of its simplicity, lower cost, and easier device integration [11,12]. Within this setting, the frontal region is of particular interest because it is closely associated with emotion regulation and depression-related neural activity while also being convenient for stable acquisition [13,14,15,16,17]. In this study, we focus on depression recognition using fixed three-channel frontal resting-state EEG recorded from Fp1, Fpz, and Fp2. This configuration covers the frontal midline and bilateral frontal areas, preserves limited yet potentially useful frontal spatial information, and is compatible with simplified forehead EEG devices. This sensing constraint affects not only hardware simplicity but also the corresponding representation learning and temporal modeling requirements. Accordingly, the aim of this study is to improve modeling performance under this fixed three-channel setting rather than to compare different channel configurations.

Despite its practical relevance, depression recognition using fixed three-channel frontal EEG remains challenging. First, temporal- and frequency-domain information has not been jointly exploited in a sufficiently integrated manner in many existing few-channel studies [18,19]. Second, when spatial observations are restricted, the model must rely more heavily on temporal discriminative patterns in EEG sequences, yet adequate and efficient long-range temporal modeling remains difficult under few-channel conditions [20,21]. Third, adaptive decision-stage mapping for heterogeneous fused representations has received relatively limited attention, although different feature sources may contribute unequally when discriminative information is constrained. As a result, the limited information available in few-channel frontal EEG may not be fully utilized.

**Figure 1:**
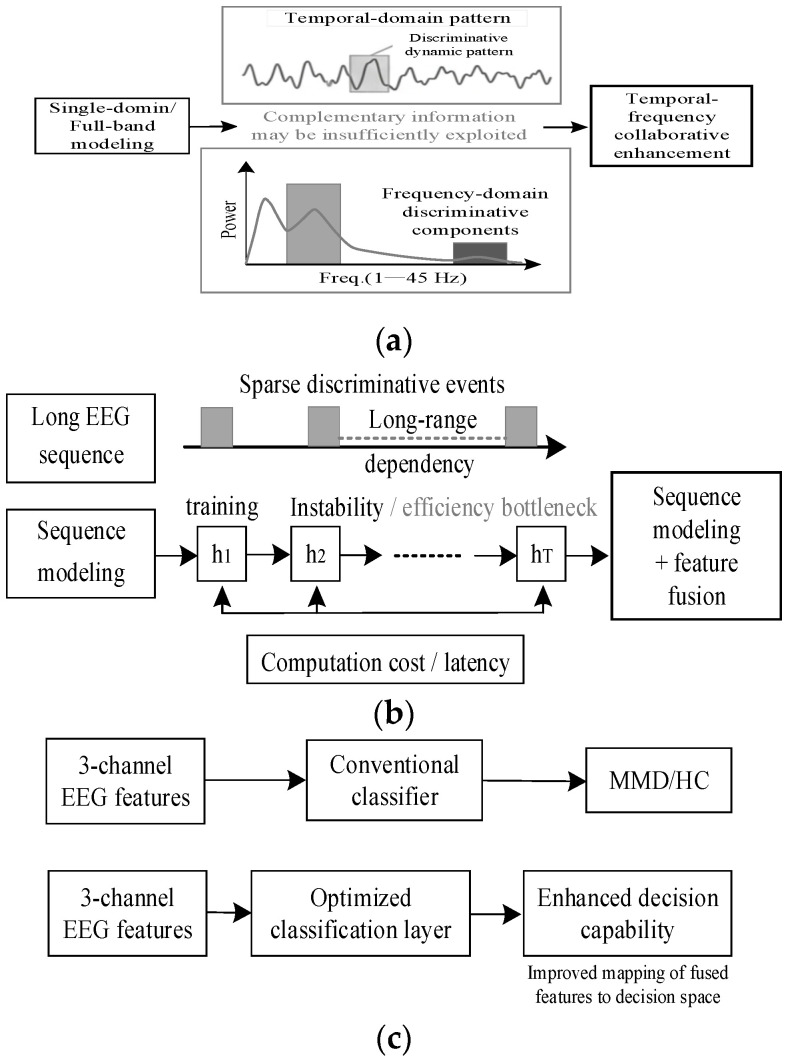
Schematic illustration of the three main challenges in few-channel EEG-based depression recognition and the corresponding design motivations of the proposed DEP-TFDualNet framework: (**a**) incomplete joint exploitation of temporal- and frequency-domain information; (**b**) inadequate long-range temporal modeling under spatially constrained observations; and (**c**) limited adaptive decision-stage mapping of heterogeneous fused representations.

To address these challenges, we propose DEP-TFDualNet, a deep learning framework tailored to fixed three-channel frontal resting-state EEG for depression recognition in acquisition-constrained settings. The framework integrates a multi-scale front end with a dual-domain attention mechanism to jointly refine temporal-domain and discrete cosine transform (DCT)-based frequency-domain representations for temporal–frequency fusion. It further employs an IndRNNv2-based temporal modeling backbone derived from the independent recurrent neural network (IndRNN) architecture to enhance long-range temporal dependency learning under spatially constrained observations. In the final stage, low-order statistical features are fused with deep representations to provide complementary global information, followed by a Kolmogorov–Arnold Network (KAN)-based nonlinear projection and linear classification as an adaptive decision-stage mapping strategy for heterogeneous fused representations. Figure 1 provides a conceptual illustration of the three main challenges in few-channel EEG-based depression recognition and the corresponding design motivations of the proposed framework.

The main contributions of this study are summarized as follows:

1. We propose DEP-TFDualNet, a deep learning framework for depression recognition from fixed three-channel frontal resting-state EEG, targeting acquisition-constrained settings relevant to simplified and potentially portable EEG-assisted screening.
2. The framework combines multi-scale convolution with dual-domain attention to jointly refine temporal-domain and DCT-based frequency-domain representations, and employs an IndRNNv2-based temporal modeling backbone to enhance long-range dependency learning under limited spatial observations.
3. A decision-stage feature integration strategy is further introduced by fusing low-order statistical descriptors with learned deep representations and applying a KAN-based nonlinear projection layer. The resulting framework is systematically evaluated on the public three-channel frontal EEG subset of MODMA using subject-level cross-validation, comparative experiments, ablation studies, and uncertainty- and stability-oriented analyses.

The remainder of this paper is organized as follows. Section 2 reviews related work. Section 3 describes the dataset, quality control procedure, input construction, and the proposed method. Section 4 presents the experimental results. Section 5 discusses the findings and limitations. Section 6 concludes the paper and outlines future work.

## 2. Related Work

To position the proposed method in the fixed three-channel frontal EEG setting, related work is reviewed from three perspectives: the transition from multi-channel to few-channel EEG for depression recognition, the use of frontal few-channel EEG for practical assessment, and deep learning approaches for representation learning, temporal modeling, and decision-stage design. Based on this review, the main limitations of existing studies under few-channel frontal EEG conditions are summarized.

### 2.1. From Multi-Channel EEG to Few-Channel Depression Recognition

Early studies on EEG-based depression recognition predominantly relied on multi-channel recordings, which provide relatively rich spatial information for feature extraction and classification. With the development of deep learning, convolutional neural networks (CNNs), recurrent neural networks (RNNs), and hybrid architectures have been increasingly introduced into this setting and have shown promising performance [20,21,22]. These studies suggest that deep models can capture nonlinear EEG characteristics related to depression more effectively than purely handcrafted pipelines in many classification settings.

However, multi-channel EEG also presents practical challenges. Although a larger number of channels provides richer spatial information, it usually requires more complex hardware, longer preparation time, and higher deployment cost, which limit its practicality in portable or low-resource screening scenarios [11,23]. In addition, previous reviews have pointed out persistent limitations related to sample size, dataset composition, and model generalizability in EEG-based depression recognition [7]. These factors further constrain the robustness and real-world transferability of multi-channel solutions.

As a result, recent studies have increasingly shifted toward few-channel EEG. Existing evidence suggests that reducing the number of electrodes does not necessarily cause a substantial loss of classification performance when informative channels are properly selected [11,12,23,24]. Simplified configurations, including three-channel frontal EEG and even single-channel EEG, have shown preliminary feasibility for depression recognition [25,26,27,28,29]. These findings indicate that few-channel EEG is not merely a reduced form of multi-channel acquisition, but also a practically meaningful direction for low-cost and simplified screening. At the same time, limited spatial observations place greater demand on effective modeling of temporal, spectral, and global signal characteristics.

### 2.2. Frontal Few-Channel EEG for Practical Depression Assessment

Among few-channel settings, the frontal region has attracted increasing attention because of its relevance to emotion regulation and depression-related neural activity, as well as its practical convenience for signal acquisition [13,14,15,16,17,30]. Frontal EEG markers, such as alpha asymmetry, have been widely associated with emotion- and depression-related differences [13,17,31]. At the same time, low-density frontal EEG is more compatible with portable devices, wearable systems, home-based monitoring, and other simplified acquisition scenarios [11,16], making it attractive for practical and resource-constrained assessment.

In this context, compact frontal configurations covering bilateral and midline regions, especially Fp1, Fpz, and Fp2, are of particular interest [28,29]. Such configurations provide a practical balance among signal availability, system simplicity, and deployment potential. Existing studies have provided preliminary evidence for their feasibility. For example, Ref. [28] used EEG from Fp1, Fpz, and Fp2, combined handcrafted linear and nonlinear features with a support vector machine (SVM) classifier, and achieved an accuracy of 72.25% in distinguishing patients with depression from healthy controls. Ref. [29] further used a portable three-electrode device with the same frontal configuration to collect resting-state eyes-closed EEG and reported an average accuracy of 83.07% using SVM. In addition, some single-channel studies have indirectly supported the usefulness of the frontal region under simplified configurations [27].

Overall, frontal few-channel EEG has become an important research setting because it combines neurophysiological relevance with application feasibility. Nevertheless, most existing work in this setting still relies mainly on handcrafted features and conventional classifiers. End-to-end deep-learning-based modeling specifically tailored to the fixed Fp1-Fpz-Fp2 resting-state configuration remains relatively limited, particularly with respect to temporal–frequency representation learning, long-range temporal dependency modeling, and adaptive decision-stage design.

### 2.3. Deep Learning Methods for Few-Channel EEG-Based Depression Recognition

#### 2.3.1. Dual-Domain Representation Learning for EEG Signals

EEG signals contain both temporal dynamics and spectral structure, and both temporal- and frequency-domain information may provide useful cues for depression recognition [8,9,19]. Existing studies generally follow two representation strategies. One uses raw EEG sequences as input and applies CNN-based models to learn temporal patterns directly [20,22]. The other constructs frequency-related representations, such as band power, power spectral density, differential entropy, or transformed spectral features, to emphasize rhythm-related differences [24,32,33]. In general, temporal signals preserve more complete dynamic information, whereas frequency-domain features are often useful for characterizing rhythm-related abnormalities.

Some studies have attempted to combine multiple EEG representations or model different frequency bands using branch-based frameworks [8,9,27]. However, the joint use of temporal- and frequency-domain information remains limited, particularly in few-channel settings. In many studies, either temporal sequences or frequency-related descriptors are emphasized as the dominant representation, while explicit interaction between the two domains is not deeply modeled. Even when multi-branch designs are adopted, fusion is often implemented in a relatively direct manner [8,9,22,34]. This issue is particularly relevant in fixed few-channel frontal EEG, where the loss of spatial diversity increases the importance of efficiently integrating complementary temporal and spectral information. Therefore, more effective temporal–frequency fusion remains an important issue in few-channel frontal EEG-based depression recognition.

#### 2.3.2. Temporal Dependency Modeling Under Long-Sequence Constraints

Temporal modeling is another important factor in EEG-based depression recognition because discriminative patterns may be sparse and distributed across relatively long signal segments. To capture such dependencies, previous studies have introduced RNNs, long short-term memory (LSTM) networks, gated recurrent units (GRUs), hybrid CNN-RNN structures, and attention-based models [8,9,10,20,21]. For example, CNN-LSTM architectures have been used to combine local pattern extraction with sequence dependency modeling and have shown favorable performance [20]. More recently, transformer- and attention-based architectures have also been explored for EEG analysis because of their ability to model global dependencies.

Nevertheless, current temporal modeling strategies still have limitations. CNN-based models are effective for local pattern extraction but are less suitable for capturing long-range temporal dependencies [20,21,22,34,35]. Recurrent structures can explicitly model sequential information, but they often increase training cost and reduce efficiency for long inputs [20,21]. Attention-based and transformer-style methods provide stronger global modeling capability, but their computational cost is usually higher, and their relative effectiveness under small-sample, few-channel conditions remains task dependent [8,9,10]. These challenges become more critical in few-channel EEG, where limited spatial information makes the model more dependent on temporal cues. As a result, adequate and efficient long-range temporal modeling has not been fully explored in fixed frontal few-channel EEG-based depression recognition.

#### 2.3.3. Decision-Stage Design for Few-Channel EEG

Compared with front-end feature extraction and backbone design, decision-stage design has received comparatively less dedicated analysis in EEG-based depression recognition studies. In many deep learning frameworks, learned or fused representations are mapped to class labels through standard fully connected layers [8,10,20,21,22,32,34,35]. Although such designs are widely used, their flexibility may be limited when the input consists of heterogeneous representations derived from temporal features, frequency-domain information, and lightweight statistical descriptors. This issue may be more relevant under few-channel conditions, where discriminative information is constrained and different feature sources may contribute unequally. Therefore, more adaptive decision-stage mapping for heterogeneous fused representations warrants further exploration in few-channel EEG-based depression recognition, especially when different fusion components may contribute differently and should be empirically distinguished.

### 2.4. Summary of Limitations in Existing Studies

In summary, existing studies support the feasibility of frontal few-channel EEG for depression recognition, but systematic modeling tailored to fixed three-channel frontal resting-state EEG remains limited. Three limitations are particularly relevant in this setting. First, temporal- and frequency-domain information has not yet been jointly exploited in a sufficiently integrated manner in many few-channel studies. Second, adequate and efficient long-range temporal modeling remains insufficient under spatially constrained input conditions, where the model must rely more heavily on temporal structure. Third, adaptive decision-stage mapping for heterogeneous fused representations has received relatively limited attention. These limitations motivate the proposed DEP-TFDualNet framework, which is designed to better utilize limited discriminative information in three-channel frontal EEG through temporal–frequency fusion, enhanced temporal dependency modeling, and adaptive decision-stage integration.

## 3. Materials and Methods

### 3.1. Dataset and Subjects

**Figure 2:**
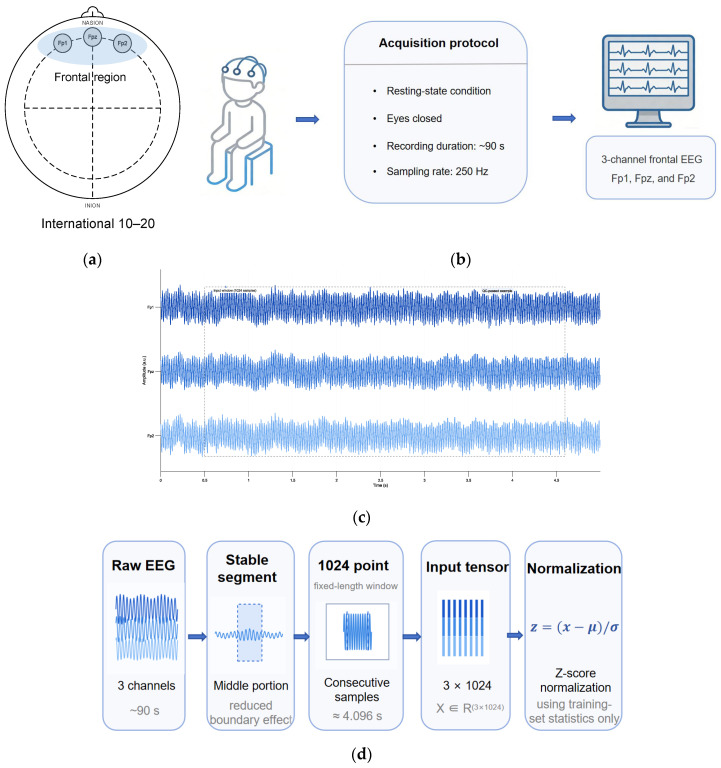
Overview of the MODMA three-channel frontal EEG subset and the input construction procedure: (**a**) the frontal three-electrode configuration Fp1, Fpz, and Fp2 under the international 10−20 system; (**b**) EEG acquisition using a wearable three-electrode device under resting-state, eyes-closed conditions, with a recording duration of approximately 90 s and a sampling rate of 250 Hz; (**c**) a representative example of a quality-controlled three-channel EEG waveform; (**d**) selection of one continuous 1024-point segment from each QC-passed recording, preferentially from the middle portion of the recording, to form a 3 × 1024 input matrix, followed by Z-score normalization using training-set statistics only.

This study used the publicly available three-channel frontal EEG subset of the Multimodal Open Dataset for Mental Disorder Analysis (MODMA) [36]. According to the dataset description, EEG signals in this subset were acquired using a wearable three-electrode device, with electrode placement following the international 10–20 system [37]. The three electrodes were positioned at the frontal sites Fp1, Fpz, and Fp2. During acquisition, subjects remained in a relatively quiet environment and underwent resting-state EEG recording with their eyes closed. Each recording lasted approximately 90 s and was sampled at 250 Hz. An overview of the dataset and the input construction procedure is shown in Figure 2.

According to the original dataset labels, the initial cohort consisted of 55 subjects, including 26 patients with major depressive disorder (MDD) and 29 healthy controls (HC). All data used in this study were obtained from the publicly available MODMA dataset, and no additional subject recruitment or new data collection was performed. According to the original MODMA report, the study protocol was approved by the Ethics Committee of the Second Hospital of Lanzhou University (CRRC-IEC-RF-SC-005-01) and complied with the Declaration of Helsinki [36].

### 3.2. Sample Selection and Quality Control

Because EEG signals, particularly frontal recordings, are highly susceptible to contamination from eye movements, blinking, and other artifacts [38], an additional recording-level quality control (QC) procedure was performed on the raw recordings to ensure that each included subject provided at least one stable analysis segment suitable for subject-level modeling. The purpose of this QC step was not to optimize classification difficulty, but to exclude recordings with severe abnormalities that precluded reliable extraction of a continuous 1024-point segment for subsequent analysis. The QC procedure combined manual visual inspection with predefined rule-based criteria and was conducted without reference to diagnostic labels, model outputs, or downstream classification results.

A recording was judged to be of low quality if any of the following conditions was present: (1) persistent baseline drift, extreme amplitude offset, or abnormally high-amplitude segments, such that no stable 1024-point segment could be obtained; (2) obvious flat segments or channel interruptions indicating signal loss; (3) abrupt transient pulses with waveform morphology inconsistent with physiological rhythms; or (4) contamination by eye movement, motion, or poor-contact artifacts over most of the recording.

**Figure 3:**
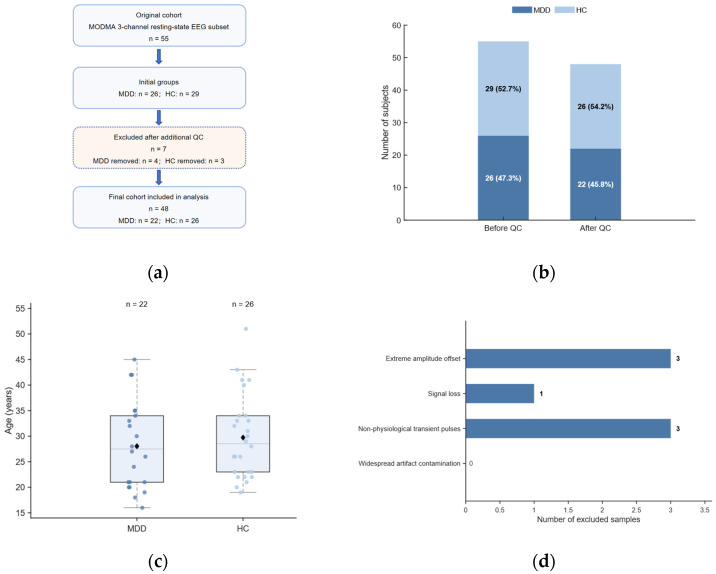
Overview of sample selection and characteristics of the final included cohort: (**a**) flowchart of sample screening. The original cohort contained 55 subjects, including 26 with MDD and 29 HCs. After quality control, seven subjects were excluded, resulting in a final cohort of 48 subjects, including 22 with MDD and 26 HCs; (**b**) group-wise sample counts before and after QC; (**c**) age distribution of the final included subjects; (**d**) main reasons for sample exclusion identified during QC.

QC was conducted before data partitioning because it served as a recording-eligibility screening step and was performed without reference to diagnostic labels, model outputs, or downstream classification results. The QC assessment was independently performed by two researchers, and disagreements were resolved through discussion until consensus was reached. After the additional QC procedure, seven subjects were excluded, including four from the MDD group and three from the HC group. The final analysis therefore included 48 subjects, comprising 22 patients with MDD and 26 HC subjects. Regarding the primary exclusion reasons, three samples were excluded because of extreme amplitude offset, one because of signal loss, and three because of non-physiological transient pulses. Although widespread artifact contamination was considered during QC, it was not the primary exclusion reason for any sample. The sample selection procedure and the distribution of exclusion reasons are shown in Figure 3.

The age distributions of the included subjects are shown in Figure 3c. Based on the final demographic statistics, the mean age was 28.05 ± 8.40 years in the MDD group and 29.73 ± 8.24 years in the HC group, with no significant between-group difference (t = −0.70, *p* = 0.49). Regarding sex distribution, the MDD group included 11 males and 11 females, whereas the HC group included 16 males and 10 females, also with no significant between-group difference (χ^2^ = 0.64, *p* = 0.42). Detailed demographic information is presented in Table 1.

### 3.3. Input Construction and Normalization

After quality control, only one continuous EEG segment was retained from each subject as the model input. This design avoided potential information leakage that could arise if multiple segments from the same subject entered different data partitions and made the evaluation more consistent with realistic subject-level application scenarios. Accordingly, each subject contributed exactly one input sample to model training and evaluation. Although this strategy does not use the full recording duration, it was intentionally adopted as a conservative design choice to avoid artificially inflating the effective sample number through within-subject duplication in the small-sample setting.

From each QC-passed recording, one continuous 1024-point segment without obvious baseline drift, signal interruption, or non-physiological transient pulses was selected as the model input. Segment selection followed a fixed recording order rule rather than diagnostic labels, model performance, or downstream classification results. The search started from the central portion of the recording to reduce potential nonstationary effects near the beginning and end. If the candidate interval in that portion did not meet the predefined quality criteria, the search was extended to adjacent temporal regions until one eligible segment was identified. The first eligible 1024-point interval identified under this rule was retained, and no alternative segment was selected based on label information or model behavior.

Finally, one continuous segment of 1024 sampling points was retained for each subject as the model input. At a sampling rate of 250 Hz, 1024 points correspond to approximately 4.096 s. This segment length was considered sufficient to cover multiple cycles of typical EEG rhythms while controlling input size and maintaining a consistent sample length under the small-sample setting. It was also computationally convenient for subsequent frequency-domain transformation.

To reduce the effect of inter-subject amplitude-scale differences and improve training stability, the input data were standardized using Z-score normalization [39,40]. For each cross-validation fold and for each channel, the mean $\mu_{c}$ and standard deviation $\sigma_{c}$ were computed from the training set only and then applied unchanged to the corresponding training, validation, and test samples:

(1) $$X_{\mathrm{norm}}^{\left(c\right)}=\frac{X_{c}-\mu_{c}}{\sigma_{c}},$$

where $X_{c}$ denotes the original signal of channel c, $X_{\mathrm{norm}}^{\left(c\right)}$ denotes the normalized signal, and $\mu_{c}$ and $\sigma_{c}$ denote the mean and standard deviation of channel c estimated from the training set of the corresponding cross-validation fold.

### 3.4. DEP-TFDualNet Architecture

**Figure 4:**
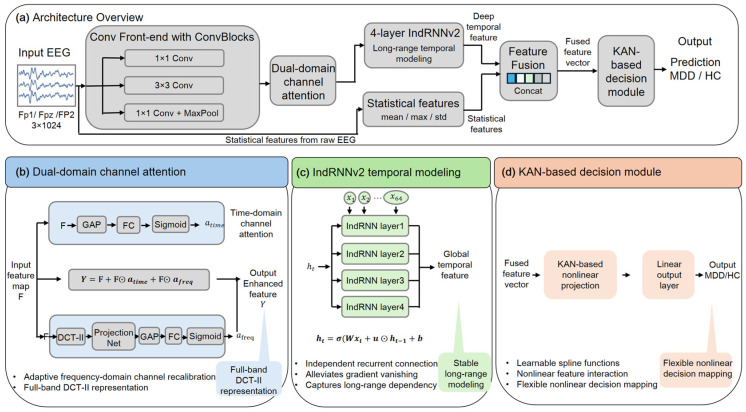
Overview of the proposed DEP-TFDualNet framework: (**a**) overall architecture of DEP-TFDualNet. A three-channel frontal EEG segment is processed by a hierarchical convolutional front end with ConvBlocks and dual-domain channel attention, followed by a four-layer IndRNNv2 for temporal modeling. Statistical descriptors extracted from the raw EEG are concatenated with the learned deep temporal representation, and the fused feature vector is then passed through a KAN-based nonlinear projection layer and a linear classifier for final prediction; (**b**) dual-domain channel attention module, which recalibrates feature responses in the time domain and the DCT-transformed frequency domain; (**c**) four-layer IndRNNv2 module for long-range temporal dependency modeling; (**d**) decision-stage adaptation using KAN-based nonlinear projection and linear output mapping.

The overall architecture of DEP-TFDualNet is illustrated in Figure 4. Using the subject-level input constructed as described in Section 3.3, DEP-TFDualNet takes one preprocessed and normalized frontal EEG segment ${X\in R}^{C\times L}$ from each subject as input, where L = 1024 is the sequence length and C = 3 corresponds to the frontal channels Fp1, Fpz, and Fp2. In implementation, the convolutional front end operates on batched tensors of shape $R^{B\times C\times L}$, where B denotes the batch size. The proposed framework consists of three stages: (1) front-end enhancement using multi-scale convolution and dual-domain channel attention; (2) temporal dependency modeling using a stacked independent recurrent neural network backbone together with low-order statistical feature fusion; and (3) decision-stage adaptation using a KAN-based nonlinear projection layer followed by a linear classifier.

This architecture was developed for the fixed three-channel frontal EEG setting considered in this study. Because spatial information is inherently limited in this configuration, the model was designed to extract and integrate discriminative temporal and spectral cues from the sequence itself. Specifically, the input EEG is first processed by a convolutional front end that progressively encodes local temporal patterns while reducing temporal resolution. Within this front end, ConvBlocks with multi-branch convolution and dual-domain channel attention perform temporal–frequency fusion by refining feature representations in both the time domain and the DCT-transformed frequency domain. The resulting compact feature sequence is then fed into a four-layer IndRNNv2 to capture higher-level temporal dependencies. In parallel, lightweight statistical descriptors are extracted directly from the raw EEG and concatenated with the learned deep temporal representation to provide complementary global signal information. Finally, the fused representation is mapped to the depression status label through a KAN-based nonlinear projection module and a linear output layer.

#### 3.4.1. Front-End Enhancement with Multi-Scale Convolution and Dual-Domain Channel Attention

Under the fixed three-channel frontal EEG setting, spatial information is inherently limited. The front end of DEP-TFDualNet therefore focuses on enhancing discriminative temporal patterns through a hierarchical convolutional encoder combined with dual-domain channel attention.

The front end begins with a hierarchy of one-dimensional convolutional stages. An initial strided convolution expands the input from 3 channels to 64 feature channels while reducing the temporal length by a factor of 2. This is followed by three additional strided convolutional stages that progressively increase the feature dimension from 64 to 128, 256, and 512, respectively, while further reducing the temporal resolution. As a result, the original 1024-point input sequence is transformed into a compact high-level feature sequence of length 64 with 512 feature channels. Each strided convolution is followed by InstanceNorm1d and the sigmoid linear unit (SiLU) activation function to improve feature normalization and nonlinear representation capacity under inter-subject variability [41,42,43].

To strengthen local representation learning at each stage, a ConvBlock is inserted after every strided convolution. Each ConvBlock adopts a three-branch parallel one-dimensional design to capture local patterns at different effective receptive scales. The first branch uses a convolution with kernel size 1 to perform channel projection and local recalibration. The second branch uses a convolution with kernel size 3 to capture short-range temporal dynamics. The third branch combines a convolution with kernel size 1 and max pooling to aggregate information over a relatively broader local temporal context. All branches are followed by InstanceNorm1d, and their outputs are fused by element-wise summation. When the input and output dimensions are matched, the block includes an identity mapping from the input to facilitate stable information propagation. After fusion, the resulting feature map is further enhanced by a dual-domain channel attention module and then passed through a SiLU activation.

To further improve representation learning under the few-channel setting, a dual-domain channel attention module is introduced to recalibrate the fused feature map in both the time domain and the DCT-transformed frequency domain. Let the input feature map to the attention module be denoted by ${F\in R}^{C_{f}\times L_{f}}$, where $C_{f}$ is the number of feature channels and $L_{f}$ is the temporal length at the current stage. In the hierarchical front end, $L_{f}$ varies with stage because the temporal resolution is progressively reduced by strided convolution; for the input sequence of length L = 1024, the stage-wise temporal lengths are 512, 256, 128, and 64, respectively.

In the time-domain branch, global average pooling is first applied along the temporal dimension to obtain a compact channel descriptor. A two-layer fully connected gating network with sigmoid activation is then used to generate the time-domain channel attention vector $a_{\mathrm{time}}{\in R}^{C_{f}}$:

(2) $$a_{\mathrm{time}}=\sigma \left(W_{t_{2}}\delta (W_{t_{1}}\mathrm{GAP}\left(F\right))\right),$$

where $GAP\left(\cdot \right)$ denotes global average pooling over the temporal dimension, $W_{t_{1}}$ and $W_{t_{2}}$ are learnable projection matrices, $\delta \left(\cdot \right)$ denotes the rectified linear unit (ReLU) activation, and $\sigma \left(\cdot \right)$ denotes the sigmoid function.

Because depression-related EEG differences may also be reflected in spectral composition, a complementary frequency-domain branch is constructed using DCT-II [44,45]. DCT-II provides a compact real-valued frequency representation and can be efficiently applied along the temporal dimension of each feature channel. In the present framework, it is applied to the full temporal axis at each stage, without handcrafted frequency-band decomposition, coefficient truncation, or explicit low-frequency selection.

Specifically, DCT-II is applied independently to each feature channel of F along the temporal dimension:

(3) $$F_{c,k}^{dct}\left[k\right]=\omega_{k}\sum_{n=0}^{L_{f}-1}F_{c,n}\;cos\;\left[\frac{\pi }{L_{f}}\left(n+\frac{1}{2}\right)k\right],$$

where $F_{c,n}$ denotes the value of the c-th feature channel at temporal index n,k is the DCT frequency index, and $\omega \left(k\right)$ is the normalization coefficient.

The resulting DCT representation is then passed through a lightweight nonlinear projection module composed of two pointwise convolutions:

(4) $${\hat{F}}^{dct}=Ρ\left(F^{dct}\right),$$

where $Ρ\left(\cdot \right)$ denotes the learnable pointwise projection. Global average pooling is subsequently applied to ${\hat{F}}^{dct}$, followed by another two-layer gating network to generate the frequency-domain channel attention vector $a_{\mathrm{freq}}{\in R}^{C_{f}}$:

(5) $$a_{\mathrm{freq}}=\sigma \left(W_{f_{2}}\delta {(W}_{f_{1}}\mathrm{GAP}\left({\hat{F}}_{\mathrm{dct}}\right))\right),$$

The two channel attention responses are finally fused with the original feature map through residual enhancement:

(6) $$Y=F\;+\left(F⊙a_{\mathrm{time}}\right)+\left(F⊙a_{\mathrm{freq}}\right),$$

where ⊙ denotes channel-wise multiplication with broadcasting along the temporal dimension.

Through this design, the front end preserves the original representation while jointly emphasizing informative responses derived from the time domain and the DCT-transformed frequency domain. This is particularly beneficial in the present three-channel frontal EEG scenario, where discriminative information must be extracted mainly from temporal structure rather than rich spatial topology.

#### 3.4.2. IndRNNv2-Based Temporal Modeling and Statistical Feature Fusion

After front-end enhancement, the encoded EEG representation is further modeled to capture longer-range temporal dependencies. This step is particularly important in the fixed three-channel setting, where limited spatial diversity makes classification more dependent on temporal evolution.

Following the convolutional encoder, the input EEG segment of length 1024 has been transformed into a feature sequence of length 64 with feature dimension 512. This sequence is then fed into a four-layer stacked IndRNNv2 backbone with hidden size 512. Compared with conventional recurrent architectures such as standard RNNs and LSTMs, IndRNN-based models are more suitable for deep recurrent stacking and long-sequence dependency learning because each hidden neuron evolves independently along the temporal dimension, which alleviates optimization difficulties caused by fully coupled recurrent transitions [46,47,48]. For simplicity, the recurrent update is expressed in the standard IndRNN form, while the implementation follows the IndRNNv2 module adopted in this study. The recurrent update at time step t can be written as:

(7) $$h_{t}=\sigma \left(W\cdot x_{t}+u⊙h_{t-1}+b\right),$$

where $x_{t}$ is the input feature vector at time step t, W is the input weight matrix, u is the learnable recurrent weight vector, b is the bias term, ⊙ denotes element-wise multiplication, and $\sigma \left(\cdot \right)$ is the activation function.

In this study, the hidden state at the final time step of the top IndRNNv2 layer is taken as the deep temporal representation, denoted by ${h\in R}^{512}$. The final hidden state was used as a compact fixed-length summary of the sequence-level temporal dependency pattern. This representation summarizes the high-level temporal dependency patterns extracted from the convolutionally encoded EEG sequence.

In parallel with deep temporal modeling, a lightweight set of low-order statistical descriptors is extracted directly from the raw EEG input to preserve global signal characteristics that may not be explicitly retained by the deep sequence representation. Specifically, for each of the three EEG channels, the mean, maximum, and standard deviation are computed along the temporal dimension. These descriptors reflect the overall amplitude level, peak magnitude, and fluctuation intensity of the signal, respectively [49]. A deliberately low-dimensional statistical set was used to provide complementary global information while limiting feature redundancy and overparameterization in the small-sample setting. Concatenating the three descriptors across the three channels yields a 9-dimensional statistical feature vector ${s\in R}^{9}$.

The final subject-level representation is obtained by concatenating the deep temporal feature and the statistical feature:

(8) z=[h;s],

where ${z\in R}^{521}$ denotes the fused representation and [;] denotes vector concatenation.

This fusion strategy combines high-level temporal semantics learned by the recurrent backbone with lightweight global descriptors computed from the raw EEG, thereby preserving both abstract temporal dependency information and coarse signal-scale information. Such multi-level fusion is especially useful under the information-constrained three-channel frontal EEG setting [42,50].

#### 3.4.3. Decision-Stage Adaptation with KAN-Based Nonlinear Projection

After temporal modeling and statistical feature fusion, the integrated representation z is mapped to the final depression-status label. Because z combines heterogeneous information from two different feature sources, a more flexible decision layer is desirable for modeling their nonlinear interactions. To this end, DEP-TFDualNet employs a KAN-based nonlinear projection module [51] followed by a linear output layer for final classification.

Let the fused feature vector be ${z\in R}^{521}$. The decision module first applies a KAN-based mapping

(9) $$g_{\mathrm{KAN}}:R^{521}\to R^{512},$$

to obtain an adapted latent representation. In the present implementation, a single KAN-based projection layer maps the 521-dimensional fused feature vector to a 512-dimensional latent representation, which is then passed to a linear layer producing two output logits corresponding to HC and MDD.

Compared with a conventional multilayer perceptron, KAN replaces fixed scalar-weighted transformations with learnable univariate functional mappings, which can provide a more adaptive nonlinear transformation for heterogeneous fused features [51]. In the implementation used here, each univariate function is parameterized as a residual combination of a base activation term and a spline term:

(10) $$\phi \left(x\right)=w_{b}\mathrm{SiLU}\left(x\right)+w_{s}\mathrm{spline}\left(x\right),$$

where $w_{b}$ and $w_{s}$ are learnable weights, $\mathrm{SiLU}\left(\cdot \right)$ denotes the sigmoid linear unit activation, and $\mathrm{spline}\left(\cdot \right)$ denotes a spline-based nonlinear component implemented using cubic B-splines (spline degree = 3).

In the present framework, KAN is not treated as an independent methodological contribution, but rather as a task-oriented decision-stage adaptation module. Its role is to enhance nonlinear integration of the deep temporal and statistical representations before final classification. This design complements the preceding temporal–frequency representation learning and recurrent temporal modeling stages and is intended to provide additional flexibility in decision-stage mapping under the small-sample, few-channel EEG classification setting.

### 3.5. Experimental Settings

#### 3.5.1. Data Partition and Evaluation Protocol

To obtain a stable evaluation under the small-sample and few-channel EEG setting, subject-wise stratified five-fold cross-validation was adopted [52]. The final dataset comprised 48 subjects, including 22 subjects with MDD and 26 HCs. Data partitioning was performed strictly at the subject level, such that each subject appeared in the test set only once across the five folds. This strategy prevented subject overlap between data splits and thereby avoided information leakage. Because the total number of subjects was not divisible by five, each test fold contained 9 or 10 subjects while preserving the class proportion as closely as possible.

Within each outer fold, approximately 20% of the subjects were assigned to the test set, and the remaining subjects formed the development set. The development set was further divided, in a subject-wise stratified manner, into training and validation subsets, with approximately 20% used for validation. Within each outer fold, the training/validation split of the development set was kept fixed across all compared models to ensure a fair comparison. The validation set was used exclusively for early stopping and training monitoring and did not participate in parameter updating or final performance evaluation. The test set remained completely independent throughout the training process. All statistics used for input normalization were computed from the training set only and then applied to the corresponding validation and test sets.

Model performance was evaluated using accuracy, sensitivity, specificity, macro-F1, the area under the receiver operating characteristic curve (AUC), and the Brier score. Accuracy, sensitivity, specificity, and macro-F1 were calculated from pooled subject-level out-of-fold predicted labels across all five folds. Specifically, each subject contributed exactly one out-of-fold predicted label and one out-of-fold predicted probability obtained when that subject appeared in the held-out test fold. AUC and Brier score were computed from pooled subject-level out-of-fold predicted probabilities for the positive class (MDD). Predicted labels were assigned to the class with the higher predicted probability, which in this binary setting is equivalent to applying a fixed threshold of 0.5 to the positive-class probability. No post hoc threshold tuning was performed on the validation or test data.

Sensitivity measured the ability of the model to identify subjects with MDD, whereas specificity measured its ability to correctly identify HCs as non-MDD. Macro-F1 was defined as the unweighted average of the class-specific F1 scores for HC and MDD. The Brier score was used to evaluate probabilistic accuracy of the predicted positive-class probabilities, with lower values indicating better probabilistic performance.

To provide an estimate of uncertainty under the small-sample setting, 95% confidence intervals (CIs) were reported for DEP-TFDualNet and selected competitive deep learning baselines in the main comparative analysis. Specifically, the CIs for accuracy, sensitivity, and specificity were estimated using the Wilson interval based on pooled subject-level out-of-fold predicted labels [53], whereas the 95% CI for AUC was estimated using the DeLong method [54].

For key between-model comparisons, subject-level paired bootstrap 95% CIs were additionally estimated from pooled out-of-fold predictions. In this procedure, each subject retained paired predictions from the two compared models, and bootstrap resampling was performed at the subject level with replacement for 10,000 iterations while preserving this pairing. For each bootstrap sample, the between-model differences in accuracy, macro-F1, AUC, and Brier score were recalculated, and percentile-based 95% CIs were derived from the bootstrap distribution. This analysis was used to quantify the uncertainty of observed performance differences under the paired small-sample setting.

#### 3.5.2. Training Configuration and Hyperparameters

All deep learning experiments were implemented in PyTorch 1.12 and conducted on an NVIDIA RTX 3090 GPU (NVIDIA Corporation, Santa Clara, CA, USA) with 24 GB of memory. Model training employed the Adam optimizer [55] with $\beta_{1}=0.9$, $\beta_{2}=0.999$, and a weight decay of $1\times 10^{-4}$. The batch size was set to 16, and the maximum number of training epochs was 50.

The initial learning rate was set to $1\times 10^{-4}$, and cosine annealing was adopted for learning rate scheduling [56], with a minimum learning rate of $1\times 10^{-5}$. The loss function for deep learning models was class-weighted cross-entropy, where the class weights were determined according to the class distribution of the training subset in each fold to mitigate the effect of mild class imbalance.

Early stopping [57] was applied by monitoring the validation loss. Training was terminated if the validation loss did not improve for 10 consecutive epochs, and the model parameters corresponding to the lowest validation loss were retained for testing. For the main comparative experiments, a fixed random seed of 3047 was used to improve reproducibility. Within each cross-validation fold, model parameters were randomly reinitialized and trained independently. Except for architectural differences, all deep learning baseline models used the same optimizer settings, learning rate schedule, batch size, loss function design, and early stopping strategy to ensure a fair comparison.

For traditional machine learning baselines, each subject was represented by the same 9-dimensional low-order statistical feature vector extracted from the raw EEG by concatenating the channel-wise mean, maximum, and standard deviation across the three frontal channels (3 descriptors × 3 channels = 9 features). Classification was then performed using k-nearest neighbors (KNN) or SVM models on the resulting feature vectors. The statistical feature vectors were standardized using training set statistics within each outer fold before classifier fitting, and the same transformation was applied to the corresponding validation and test subjects. For KNN, the number of neighbors was set to k = 5. For SVM, a radial basis function (RBF) kernel was used. Both traditional machine learning baselines were trained using the full development set of each outer fold, without access to the corresponding test subjects. These traditional machine learning baselines were included as reference methods under the same low-order statistical feature setting, rather than as exhaustive reproductions of feature-engineered pipelines reported in prior studies.

#### 3.5.3. Baseline Models, Ablation Study, and Supplementary Analyses

To comprehensively evaluate DEP-TFDualNet for few-channel frontal EEG-based depression recognition, both traditional machine learning and deep learning baselines were included for comparison:

1. KNN, a k-nearest neighbors classifier used as a traditional machine learning baseline;
2. SVM, a support vector machine classifier used as a traditional machine learning baseline;
3. CNN, a convolutional neural network that extracts features from raw EEG input and performs classification through fully connected layers;
4. LSTM, which directly models EEG temporal signals using a long short-term memory network;
5. CNN-LSTM, which first extracts local temporal features using convolutional layers and then models long-range temporal dependencies using LSTM;
6. EEG-Transformer, an attention-based baseline included to provide comparison with a contemporary transformer-style EEG classifier under the same subject-wise evaluation protocol;
7. DEP-TFDualNet, the proposed full model consisting of the front-end enhancement module with multi-scale convolution and dual-domain channel attention, the IndRNNv2-based temporal modeling backbone, low-order statistical feature fusion, and a KAN-based nonlinear projection layer followed by a linear classifier for HC/MDD prediction.

All models were evaluated using the same subject-wise data partition and evaluation protocol. For deep learning models, the training configuration was kept identical as far as possible to ensure fairness.

To further investigate the contributions of the main architectural modifications introduced in this study, an extended ablation analysis was conducted using CNN-LSTM as the reference model. Specifically, the following model groups were considered: Group A, the baseline CNN-LSTM; Group B, Group A with the standard convolutional front end replaced by the proposed front-end enhancement module incorporating multi-scale convolution and dual-domain channel attention, yielding Improved CNN-LSTM; Group C, Group B with the LSTM-based temporal modeling component further replaced by IndRNNv2, yielding Improved CNN-IndRNN with a standard fully connected decision head; Group D, Group C with the final fully connected layer replaced by a KAN-based nonlinear projection module while retaining only the deep temporal representation, yielding Improved CNN-IndRNN + KAN; Group E, Group C with low-order statistical feature fusion introduced before a standard fully connected decision head, yielding Improved CNN-IndRNN + statistics + FC; and Group F, the full DEP-TFDualNet, in which both low-order statistical feature fusion and KAN-based nonlinear projection were jointly incorporated. Through this design, the contributions of front-end enhancement, improved temporal dependency modeling, KAN-based decision adaptation, and low-order statistical feature fusion could be evaluated in a more separated and interpretable manner.

To further improve interpretability, an additional channel-level contribution analysis was conducted using a leave-one-channel-out protocol on the three frontal channels (Fp1, Fpz, and Fp2). Specifically, each channel was removed in turn from the input, and the corresponding reduced-channel variant was evaluated under the same subject-wise five-fold cross-validation protocol and training configuration as the full model. The resulting performance change relative to the full three-channel model was used to assess the contribution of the removed channel. A larger performance degradation indicates that the removed channel provides more discriminative information for depression classification under the current experimental setting.

Because DEP-TFDualNet and the strongest recurrent baseline, CNN-LSTM, produced paired subject-level out-of-fold class predictions under the same cross-validation partition, the continuity-corrected McNemar test [58] was used as an auxiliary comparison of subject-level classification disagreement. McNemar’s test is appropriate in this setting because it evaluates whether two paired classifiers differ in their proportions of discordant binary outcomes on the same subjects. Given the limited sample size, this analysis was intended only as supportive evidence and does not replace metric-specific uncertainty assessment, particularly for probability-based measures such as AUC and Brier score.

To further assess robustness, repeated-run stability analysis was conducted for CNN-LSTM and DEP-TFDualNet over five independent runs with different random seeds while keeping the same subject-wise five-fold data partition fixed. Different seeds affected parameter initialization, mini-batch ordering, and stochastic optimization dynamics, whereas the subject partition remained unchanged. The training/validation split within each outer fold was also kept fixed across repeated runs, so that variability primarily reflected stochastic training effects. In each run, accuracy, sensitivity, specificity, and macro-F1 were computed from pooled subject-level out-of-fold predicted labels, whereas AUC and Brier score were computed from pooled subject-level out-of-fold predicted probabilities for the positive class. The results were summarized as mean ± standard deviation across runs.

To examine possible overfitting under the small-sample setting, epoch-wise training and validation dynamics were additionally recorded for CNN-LSTM and DEP-TFDualNet under the same fixed outer data partition. Specifically, training loss, validation loss, training accuracy, and validation accuracy were tracked across epochs within each fold and then summarized by fold-averaged curves. An accuracy-based generalization gap was further computed as the difference between training accuracy and validation accuracy in the final monitored epochs. These analyses were included as supplementary indicators of optimization behavior and overfitting risk rather than as standalone performance criteria.

In addition, the number of trainable parameters was recorded as a supplementary implementation indicator. Finally, to better characterize model behavior beyond aggregate metrics, a descriptive subject-level error analysis was performed based on pooled out-of-fold predictions to summarize the demographic distribution and probability characteristics of misclassified subjects. Because the numbers of misclassified subjects were very small, this analysis was interpreted descriptively without formal inferential testing.

## 4. Results

### 4.1. Overall Performance Comparison

DEP-TFDualNet was compared with two conventional machine learning models (KNN and SVM) and four representative deep learning models (CNN, LSTM, CNN-LSTM, and EEG-Transformer). The results are summarized in Table 2. Accuracy, sensitivity, specificity, and macro-F1 were computed from pooled subject-level out-of-fold predicted labels under five-fold cross-validation, whereas AUC and Brier score were computed from pooled subject-level out-of-fold predicted probabilities for the positive class (MDD). Predicted labels were assigned to the class with the higher predicted probability, which in this binary setting is equivalent to using a fixed threshold of 0.5. No post hoc threshold tuning was performed. To avoid redundancy, 95% CIs for accuracy, sensitivity, specificity, and AUC are reported only for DEP-TFDualNet and the two most competitive deep learning baselines, CNN-LSTM and EEG-Transformer, because these three models constituted the most relevant comparisons for the proposed method in terms of deep learning performance.

As shown in Table 2, DEP-TFDualNet achieved the best threshold-based performance among all evaluated models, with an accuracy of 85.42%, sensitivity of 81.82%, specificity of 88.46%, and macro-F1 of 85.26%. It also yielded the lowest observed Brier score (0.121) among all evaluated models. Its AUC was 0.82, which was higher than that of CNN-LSTM (0.77) and numerically comparable to that of EEG-Transformer at the reported precision. Compared with CNN-LSTM, DEP-TFDualNet improved accuracy and macro-F1 by 8.33 and 8.43 percentage points, respectively, increased AUC by 0.05, and reduced the Brier score by 0.054. Compared with EEG-Transformer, it improved accuracy and macro-F1 by 10.42 and 10.30 percentage points, respectively, reduced the Brier score by 0.048, and showed the same AUC at the reported precision. Overall, these point estimates suggest that DEP-TFDualNet provided the most favorable balance between threshold-based classification performance and probabilistic accuracy in the current dataset.

To assess the uncertainty of the between-model comparisons, subject-level paired bootstrap 95% CIs were estimated from pooled out-of-fold predictions and are reported in Table 3.

The point estimates favored DEP-TFDualNet over both CNN-LSTM and EEG-Transformer for accuracy, macro-F1, and Brier score. For AUC, the observed difference was positive relative to CNN-LSTM and zero relative to EEG-Transformer at the reported precision. However, the paired bootstrap CIs for accuracy, macro-F1, and AUC all crossed zero, indicating that these between-model differences should be interpreted cautiously in this limited cohort. For Brier score, DEP-TFDualNet showed a lower value than CNN-LSTM, and the paired bootstrap CI excluded zero (−0.103 to −0.008), supporting an advantage in probabilistic accuracy for that comparison. In contrast, the comparison with EEG-Transformer remained inconclusive because the CI for the Brier score difference crossed zero (−0.093 to 0.004). Overall, the paired analyses indicate a favorable numerical profile for DEP-TFDualNet, although larger cohorts will be needed to estimate the magnitude of the between-model differences more precisely.

**Figure 5:**
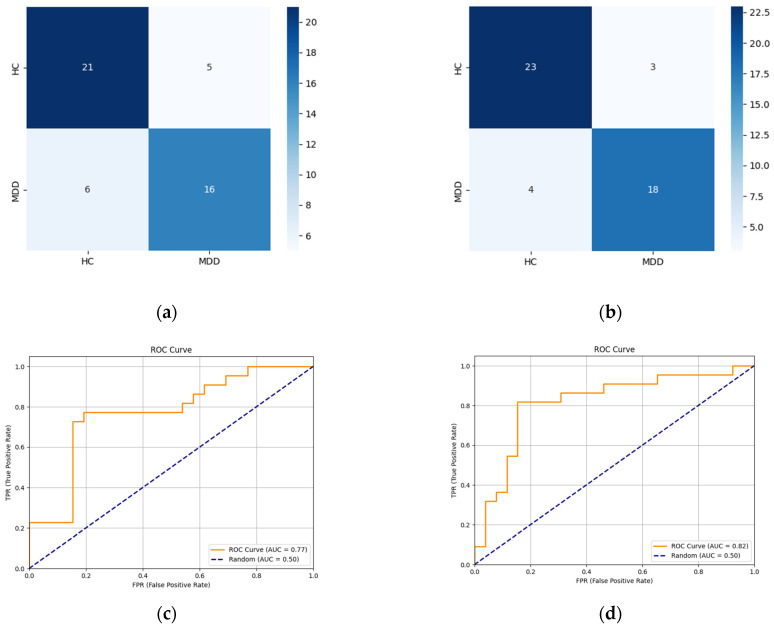
Confusion matrices based on pooled subject-level out-of-fold predicted labels and ROC curves based on pooled subject-level out-of-fold predicted probabilities for CNN-LSTM and DEP-TFDualNet under five-fold cross-validation. (**a**) CNN-LSTM confusion matrix; (**b**) DEP-TFDualNet confusion matrix; (**c**) CNN-LSTM ROC curve; and (**d**) DEP-TFDualNet ROC curve.

For visual comparison, Figure 5 presents the confusion matrices and ROC curves of CNN-LSTM and DEP-TFDualNet. CNN-LSTM was selected for focused visualization because it provided the strongest overall threshold-based baseline performance and thus served as the primary comparator in the main classification setting. EEG-Transformer was retained in the quantitative comparisons because of its competitive AUC, but was not additionally visualized here to avoid overcrowding the figure. Consistent with Table 2, DEP-TFDualNet yielded fewer misclassifications than CNN-LSTM at the fixed decision threshold. Repeated-run stability analysis is presented in Section 4.4, complementary analyses of training–validation dynamics and generalization gap are provided in Section 4.5, and the characteristics of misclassified subjects are described in Section 4.7.

### 4.2. Ablation Study

To further evaluate the contribution of the main design components in DEP-TFDualNet, a component-wise ablation study was conducted under the same five-fold cross-validation protocol. Metrics were computed in the same manner as in Section 4.1. To separately assess the effects of the KAN-based projection head and the statistical feature branch, Improved CNN-IndRNN + KAN and Improved CNN-IndRNN + statistics + FC were evaluated as two parallel variants on the same Improved CNN-IndRNN backbone. The results are summarized in Table 4.

As shown in Table 4, introducing the proposed front-end enhancement module improved accuracy from 77.08% to 79.17%, sensitivity from 72.73% to 81.82%, and macro-F1 from 76.83% to 79.13%, while reducing the Brier score from 0.175 to 0.166. Specificity decreased slightly from 80.77% to 76.92%, and AUC remained unchanged at 0.77. After replacing the LSTM-based temporal modeling module with IndRNNv2, accuracy further increased to 83.33%, specificity rose to 84.62%, macro-F1 increased to 83.22%, AUC increased from 0.77 to 0.81, and the Brier score decreased to 0.141, while sensitivity remained unchanged at 81.82%.

To separate the contributions of the classifier head and the statistical feature branch, two parallel variants were further compared on the same Improved CNN-IndRNN backbone. Relative to Improved CNN-IndRNN + FC, Improved CNN-IndRNN + KAN yielded the same threshold-based classification metrics and the same AUC at the reported precision, but reduced the Brier score from 0.141 to 0.133. In contrast, Improved CNN-IndRNN + statistics + FC increased accuracy to 85.42%, specificity to 88.46%, and macro-F1 to 85.26%, while reducing the Brier score to 0.127. Sensitivity remained 81.82%, and AUC remained 0.81 at the reported precision. The full DEP-TFDualNet integrates both the statistical feature branch and the KAN-based projection head. Compared with Improved CNN-IndRNN + statistics + FC, DEP-TFDualNet preserved the same threshold-based classification metrics while slightly increasing AUC from 0.81 to 0.82 and reducing the Brier score from 0.127 to 0.121. Compared with Improved CNN-IndRNN + KAN, it improved accuracy by 2.09 percentage points, specificity by 3.84 percentage points, and macro-F1 by 2.04 percentage points, while also yielding a lower Brier score.

To further assess the uncertainty of the small probabilistic performance differences among the key ablation variants, paired bootstrap 95% confidence intervals for selected pairwise differences in AUC and Brier score were estimated from pooled subject-level out-of-fold predictions and are reported in Table 5. Threshold-based metrics were not included in Table 5 because they were identical in two of the three selected comparisons, and the remaining threshold-based differences were already directly summarized in Table 4. For each subject, the out-of-fold predictions from the two models being compared were retained as a matched pair, and bootstrap resampling was performed at the subject level with replacement while preserving this pairing. The point estimates consistently favored the added KAN head or statistical feature branch in their respective comparisons, particularly in terms of lower Brier score. Specifically, replacing the FC head with the KAN-based projection head was associated with a Brier score reduction of 0.008, and adding the statistical-feature branch to the FC-based variant was associated with a Brier score reduction of 0.014. Relative to Improved CNN-IndRNN + statistics + FC, the full DEP-TFDualNet further reduced the Brier score by 0.006 while numerically increasing AUC by 0.01. However, all paired bootstrap 95% confidence intervals overlapped zero, indicating that these improvements should be interpreted as directional rather than definitive under the current sample size. Taken together with the component-wise trends in Table 4, these results suggest that the statistical feature branch was mainly associated with gains in threshold-based classification metrics, whereas the KAN-based projection head was mainly associated with improved probabilistic prediction quality.

### 4.3. Channel Removal Analysis

A leave-one-channel-out analysis was conducted to evaluate the effect of removing each frontal EEG channel (Fp1, Fpz, and Fp2) in DEP-TFDualNet. The full three-channel model was compared with three reduced-input variants, each excluding one channel while keeping the remaining experimental settings unchanged. The results are shown in Table 6.

As shown in Table 6, the full three-channel model achieved the highest accuracy, specificity, macro-F1, and AUC, together with the lowest Brier score, whereas all reduced-input variants showed numerically lower performance than the full model. Among them, the largest overall degradation was observed when Fpz was removed, whereas the smallest overall degradation was observed when Fp2 was removed.

To quantify the uncertainty of the channel removal effects, subject-level paired bootstrap 95% confidence intervals for selected between-model differences were estimated from the pooled out-of-fold predictions and are reported in Table 7. Across all three comparisons, the observed point estimates consistently favored the full three-channel model in terms of accuracy, AUC, and Brier score. The largest numerical deterioration relative to the full model was observed for the variant without Fpz, whereas the variant without Fp2 remained closest to the full model. However, all paired bootstrap 95% confidence intervals overlapped zero, indicating that these channel-specific differences should be interpreted cautiously under the current sample size and regarded as directional rather than definitive evidence.

### 4.4. Repeated-Run Stability Analysis

To further assess the stability of model performance with respect to training stochasticity under the small-sample setting, repeated experiments with five different random seeds were conducted for CNN-LSTM and DEP-TFDualNet while keeping the same outer subject-level five-fold partition fixed. Under this design, the repeated-run analysis primarily reflected sensitivity to stochastic training factors, including parameter initialization, mini-batch ordering, and optimization dynamics, rather than variability introduced by different data partitions. EEG-Transformer was evaluated in the main comparative experiment; however, the repeated-run analysis in this section was intentionally focused on CNN-LSTM and DEP-TFDualNet, as the primary goal was to examine whether the performance improvement of the proposed model over the main convolutional–recurrent baseline remained stable across different random initializations under the same fixed partition. The mean and standard deviation of the evaluation metrics across the five runs are summarized in Table 8.

Across repeated runs, DEP-TFDualNet consistently showed higher mean performance than CNN-LSTM across all evaluation metrics, while the standard deviations remained relatively small under the fixed partition setting. Specifically, DEP-TFDualNet achieved an average accuracy of 84.17% compared with 76.67% for CNN-LSTM, and also showed higher mean macro-F1 (83.94% vs. 76.21%), sensitivity (80.91% vs. 73.64%), specificity (86.92% vs. 79.62%), and AUC (0.81 vs. 0.76). In addition, the run-to-run variability of DEP-TFDualNet remained low across the five random seeds, with standard deviations of 2.14% for accuracy, 3.88% for sensitivity, 3.16% for specificity, 2.26% for macro-F1, and 0.03 for AUC. These findings indicate that the performance advantage of DEP-TFDualNet was generally maintained across different random initializations in the current cohort under the fixed partition protocol, suggesting that the observed improvement was not attributable to a favorable single-seed outcome alone. This repeated-run analysis was intended to examine sensitivity to stochastic training factors under a fixed data partition, rather than to provide a comprehensive assessment of variability across alternative cross-validation splits.

### 4.5. Overfitting and Training–Validation Dynamics Analysis

**Figure 6:**
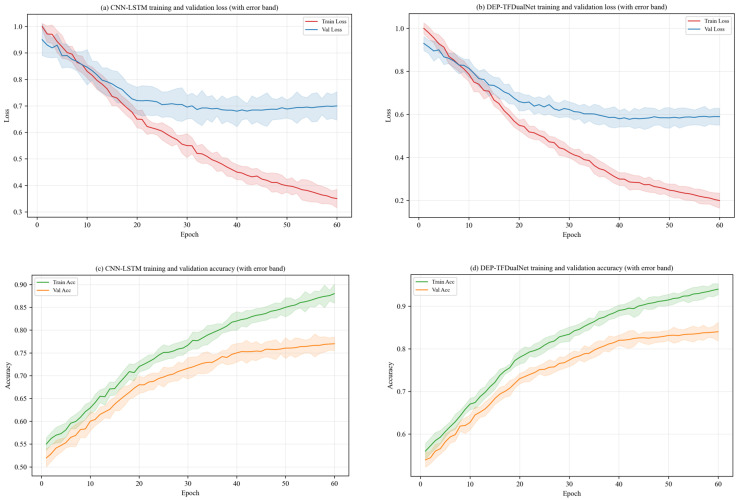
Training and validation loss and accuracy curves based on the same outer subject-level five-fold cross-validation partition for CNN-LSTM and DEP-TFDualNet. Curves represent the epoch-wise mean across the five folds, and shaded bands indicate ±1 standard deviation across folds at each epoch. (**a**) CNN-LSTM training and validation loss; (**b**) DEP-TFDualNet training and validation loss; (**c**) CNN-LSTM training and validation accuracy; and (**d**) DEP-TFDualNet training and validation accuracy.

To further examine whether the superior performance of DEP-TFDualNet may have been achieved at the cost of increased overfitting, we compared its training and validation dynamics with those of CNN-LSTM under the same outer subject-level five-fold cross-validation protocol. For each model, training loss, validation loss, training accuracy, and validation accuracy were recorded at each epoch for each fold, and the corresponding epoch-wise mean curves across the five folds are presented in Figure 6.

Figure 6 shows that both models exhibited the expected separation between training and validation performance during optimization while remaining generally convergent. For CNN-LSTM, the validation loss decreased mainly during the early-to-middle stage of training and then entered a plateau with mild fluctuation, whereas the training loss continued to decline during later epochs. A corresponding pattern was observed in the accuracy curves, where training accuracy increased steadily while validation accuracy improved more gradually and stabilized at a lower level. DEP-TFDualNet showed a broadly similar optimization pattern, but with lower validation loss and higher validation accuracy over much of the later training stage. Importantly, it did not appear to exhibit a larger train–validation separation than CNN-LSTM under the current protocol, and its validation behavior was broadly comparable by visual inspection.

This visual pattern was broadly consistent with the quantitative results summarized in Table 9. Specifically, CNN-LSTM achieved a mean training accuracy of 88.1 ± 1.6% and a mean validation accuracy of 76.8 ± 1.8%, corresponding to an accuracy-based generalization gap of 11.3 ± 1.3%, whereas DEP-TFDualNet achieved 93.8 ± 1.4% training accuracy and 84.3 ± 1.5% validation accuracy, yielding a numerically smaller accuracy-based generalization gap of 9.5 ± 1.2%. Overall, these findings do not indicate more severe overfitting in DEP-TFDualNet than in the comparator baseline under the present experimental setting. Nevertheless, this result should be interpreted with caution. A smaller train–validation gap does not exclude the possibility of overfitting, particularly given the limited cohort size and the relatively high model capacity. Accordingly, the present analysis should be viewed as supportive evidence from training dynamics and quantitative comparison rather than definitive proof of robust out-of-sample generalization.

### 4.6. Subject-Level Statistical Comparison and Model Complexity

Consistent with the subject-level comparison in Table 2 and the repeated-run stability analysis in Section 4.4, DEP-TFDualNet showed higher point estimates than CNN-LSTM across the main evaluation metrics. As a supplementary paired subject-level comparison based on the pooled out-of-fold predicted labels obtained under the same five-fold cross-validation partition, a continuity-corrected McNemar test was performed for CNN-LSTM and DEP-TFDualNet. The result did not reach statistical significance ($\chi^{2}=2.18$, p=0.15). Therefore, although DEP-TFDualNet showed a numerically favorable trend, the difference in classification outcomes should be interpreted cautiously in this limited cohort.

DEP-TFDualNet contained approximately 5.2 million trainable parameters, compared with 7.1 million for CNN-LSTM. Thus, under the present implementation, the proposed model used fewer parameters than CNN-LSTM, although its capacity remained substantial relative to the current sample size. Runtime was not treated as a formal comparison endpoint in this study because it can be substantially influenced by hardware configuration, batch size, and evaluation protocol. Therefore, the implementation-related comparison reported here was limited to parameter count as a supplementary descriptor rather than a standalone indicator of model efficiency.

### 4.7. Descriptive Analysis of Misclassified Subjects

To further characterize the error patterns of DEP-TFDualNet, a descriptive analysis was conducted on the seven misclassified subjects identified from the pooled subject-level out-of-fold predictions, including four false negatives (FNs) and three false positives (FPs). Their clinical characteristics were descriptively compared with those of correctly classified subjects, as summarized in Table 10. No formal statistical inference was performed for these subgroup comparisons because the numbers of misclassified subjects were very small.

Descriptively, FN subjects had lower mean PHQ-9 and GAD-7 scores than TP subjects, whereas FP subjects showed somewhat higher mean PSQI and LES values than TN subjects. These patterns may reflect heterogeneity among borderline or atypical cases, although no formal inference is warranted given the very small subgroup sizes. The GAD-7/PHQ-9 ratio also varied across groups, but the small number of cases precludes any firm interpretation. Overall, these observations are intended only to provide an exploratory description of potential error patterns and should be interpreted cautiously.

## 5. Discussion

In this study, DEP-TFDualNet was developed for computer-aided depression recognition using fixed three-channel frontal resting-state EEG and evaluated on the MODMA dataset. Compared with conventional machine learning models and representative deep learning baselines, DEP-TFDualNet showed the most favorable threshold-based subject-level point estimates and the lowest Brier score in the current cohort, while its AUC was higher than that of CNN-LSTM and numerically comparable to that of EEG-Transformer. Relative to CNN-LSTM, the strongest convolutional–recurrent baseline, DEP-TFDualNet also showed a consistently favorable numerical trend, and the corresponding confidence-interval analyses did not contradict this numerical pattern, although most paired confidence intervals remained wide and crossed zero under the current small-sample setting. These findings suggest that collaborative temporal–frequency modeling, strengthened temporal dependency learning, and flexible decision-stage feature integration may help improve the utilization of limited discriminative information in the few-channel frontal EEG setting. However, the continuity-corrected McNemar test did not show a statistically significant difference between DEP-TFDualNet and CNN-LSTM. At the same time, the repeated-run analysis showed a similar performance advantage under different random initializations. Therefore, the observed improvement should be interpreted cautiously and requires further validation in larger cohorts.

### 5.1. Main Findings and Possible Interpretations

The main finding of this study was that DEP-TFDualNet showed a consistently favorable classification trend relative to KNN, SVM, CNN, LSTM, and CNN-LSTM under the fixed three-channel frontal EEG setting, while also achieving the lowest observed Brier score among the evaluated models. This pattern is broadly consistent with the design objective of the proposed framework, which aimed to address several practical challenges in few-channel frontal EEG-based depression recognition, including limited spatial information, incomplete joint use of temporal- and frequency-domain information, insufficient long-range temporal modeling, and the need for more flexible integration of heterogeneous fused representations.

First, the proposed front end combined multi-scale convolution with dual-domain attention to enhance temporal patterns at different scales and improve cross-domain information utilization. Depression-related EEG abnormalities have been reported in both temporal dynamics and frequency-domain characteristics [7,19]. Relative to single-representation approaches, jointly enhancing temporal-domain and DCT-based frequency-domain features may help capture complementary discriminative information under the few-channel setting. The ablation results supported the contribution of this front-end design, although its effect was reflected mainly in improved sensitivity rather than uniform gains across all evaluation metrics. At the same time, the present study did not include dedicated interpretability analyses to determine which parts of the DCT-transformed representation were most strongly weighted by the attention mechanism. Therefore, neurophysiological interpretation should remain cautious.

Second, under the fixed three-channel frontal setting, spatial information is inherently limited, which increases the importance of temporal dependency modeling [20,28]. After the temporal modeling module was replaced from LSTM to IndRNNv2, specificity increased while sensitivity was maintained, and AUC also improved. This pattern suggests that strengthened temporal dependency modeling may help improve overall discrimination capability under spatially constrained EEG observations. This may be particularly relevant in acquisition settings where only a small number of frontal leads are available and spatial information is limited.

Third, the proposed framework adopted low-order statistical feature fusion together with a KAN-based decision-stage adaptation strategy for the final fused representation. In this design, low-order statistical features extracted from the raw EEG were concatenated with the learned deep temporal representation to provide complementary low-complexity global information, and KAN was then used to provide a flexible nonlinear mapping over the heterogeneous fused features. The ablation results suggested that these two components played different roles. Relative to the Improved CNN-IndRNN + FC variant, the KAN-based projection head did not materially change threshold-based classification metrics at the reported precision, but was associated with a lower Brier score, suggesting a possible benefit in probabilistic prediction quality. In contrast, the statistical feature branch was more clearly associated with improvements in accuracy, specificity, and macro-F1. At the same time, the lower performance of KNN and SVM using the same 9-dimensional statistical descriptors alone suggests that these handcrafted summaries were useful yet insufficient by themselves, and that their benefit was realized mainly when integrated with the learned deep representation. Nevertheless, because the current cohort was small and the paired confidence intervals for the key ablation comparisons crossed zero, the magnitude of these component-level contributions remains uncertain and should be interpreted as directional rather than definitive.

As a supplementary input-level analysis, the channel removal analysis in Section 4.3 provided a preliminary assessment of channel-specific contributions under the current fixed three-channel frontal setting. Numerically, model performance decreased most after removing Fpz, whereas removing Fp2 produced the smallest performance reduction, suggesting that the three frontal channels may not contribute equally to the final prediction. One possible interpretation is that Fpz may carry relatively more useful information for the present task, while Fp1 and Fp2 still provide complementary lateral frontal signals. However, because the corresponding confidence intervals remained wide and crossed zero, these observations should be interpreted as exploratory and directional rather than as definitive evidence of channel importance.

The descriptive analysis of misclassified subjects provided some preliminary clues regarding difficult-to-classify cases. False-negative subjects showed lower PHQ-9 and GAD-7 scores than true-positive subjects, whereas false-positive subjects showed somewhat less favorable sleep- and stress-related profiles than true-negative subjects. These patterns may reflect phenotypic heterogeneity and the challenge of classifying boundary cases using only limited frontal EEG information [59,60]. In addition, variability in the GAD-7/PHQ-9 ratio among misclassified subjects may be relevant to anxiety–depression overlap, which is common in clinical practice [61]. However, because the number of misclassified cases was very small and no formal statistical inference was performed, these observations should be regarded as exploratory and hypothesis-generating only.

From an application perspective, these findings are relevant to simplified and portable EEG-assisted screening scenarios, in which only a small number of frontal electrodes can be practically deployed. In this sense, the present study provides preliminary support for the feasibility of depression-related discrimination under acquisition-constrained EEG settings, although further validation is required before clinical translation can be considered.

### 5.2. Limitations and Future Directions

Several limitations should be acknowledged. First, the sample size was relatively small, and validation was conducted on a single public dataset. Only 48 subjects were included in the final analysis, which may limit statistical power and generalizability. In addition, although repeated-run experiments suggested relatively stable performance under different random initializations, the subject-level data partition was fixed across runs. Therefore, the stability analysis mainly reflected robustness to training stochasticity under a controlled partition protocol rather than variation across different subject partitions. Future studies should perform external validation on larger, multi-center, and cross-device datasets and should further examine robustness under multiple independent partition schemes.

Second, although collaborative temporal–frequency modeling improved classification performance, model interpretability remains limited. The relative contributions of specific temporal regions, DCT-transformed feature responses, and feature branches to the final decision are still unclear. Although the channel removal analysis provided an initial view of channel-specific contribution patterns, it remained a coarse analysis under the current fixed three-channel setting and did not establish definitive channel importance or an optimal channel configuration. In addition, the current quality control and segment selection procedures still involved manual inspection, which may limit scalability, reproducibility, and comparability with studies using more automated artifact handling pipelines. Future work should combine attention visualization, feature attribution analysis, and branch-level or component-level ablation to better clarify the roles of different modules and features. More systematic channel selection or channel importance analyses, together with standardized automated preprocessing and quality control strategies, are also needed to improve interpretability, scalability, and reproducibility.

Third, the present subject-level design retained only one continuous 1024-point segment from each recording. This conservative strategy helped avoid within-subject duplication and information leakage, but it may underuse the full recording and does not address robustness to alternative leakage-safe segment selection or aggregation strategies. Future work should examine whether similar findings hold under repeated segment sampling or subject-level aggregation across multiple non-overlapping segments.

Fourth, the present task was formulated as an offline binary classification problem between MDD and HC. Although this setting is appropriate for methodological validation, it still differs from real clinical screening scenarios, where symptom severity, psychiatric comorbidity, medication status, and population heterogeneity may be substantially more complex. Future work may incorporate richer symptom dimensions, clinical scale information, or multimodal physiological signals and further evaluate the framework in prospective screening or clinically realistic deployment settings.

Finally, although the proposed framework had fewer trainable parameters than CNN-LSTM, runtime-related implementation characteristics were not emphasized in this study because such measurements depend strongly on hardware configuration, batch size, and testing protocol. Future studies may include more standardized efficiency benchmarking to better assess the deployment potential of the method in portable EEG-assisted applications.

## 6. Conclusions

This study proposed DEP-TFDualNet, a collaborative framework for computer-aided depression recognition using fixed three-channel frontal resting-state EEG. On the MODMA dataset, DEP-TFDualNet achieved 85.42% accuracy, 81.82% sensitivity, 88.46% specificity, 85.26% macro-F1, an AUC of 0.82, and a Brier score of 0.121, showing a favorable overall balance between subject-level classification performance and probabilistic accuracy among the compared models. Relative to CNN-LSTM, it improved accuracy and macro-F1 by 8.33 and 8.43 percentage points, respectively, increased AUC by 0.05, and reduced the Brier score by 0.054. These findings suggest that temporal–frequency enhancement, strengthened temporal dependency modeling, and flexible decision-stage feature integration may improve the use of limited discriminative information in few-channel frontal EEG. However, given the limited sample size, lack of external validation, and non-significant McNemar test result versus CNN-LSTM, the findings should be interpreted cautiously. Overall, this study provides preliminary support for depression recognition using simplified frontal EEG and may inform future work on portable EEG-assisted screening.

## Figures and Tables

**Table 1:** Demographic characteristics of the included subjects.

Variable	MDD (*n* = 22)	HC (*n* = 26)	Statistic	*p*-Value
Age (years, mean ± SD)	28.05 ± 8.40	29.73 ± 8.24	t = −0.70	0.49
Sex (male/female)	11/11	16/10	χ^2^ = 0.64	0.42

**Table 2:** Performance comparison of DEP-TFDualNet and baseline models.

Model	Accuracy (%)	Sensitivity (%)	Specificity (%)	Macro-F1 (%)	AUC	Brier Score
KNN	68.75	63.64	73.08	68.41	0.70	0.223
SVM	70.83	59.09	80.77	70.00	0.65	0.232
CNN	72.92	72.73	73.08	72.81	0.68	0.207
LSTM	75.00	72.73	76.92	74.83	0.69	0.199
EEG-Transformer	75.00 (61.22–85.09)	77.27 (56.57–89.87)	73.08 (53.91–86.29)	74.96	0.82 (0.70–0.94)	0.169
CNN-LSTM	77.08 (63.46–86.68)	72.73 (51.84–86.86)	80.77 (62.12–91.48)	76.83	0.77 (0.63–0.91)	0.175
DEP-TFDualNet	85.42 (72.83–92.75)	81.82 (61.51–92.73)	88.46 (71.02–96.00)	85.26	0.82 (0.70–0.94)	0.121

Note: Accuracy, sensitivity, specificity, and macro-F1 were computed from pooled subject-level out-of-fold predicted labels under five-fold cross-validation, whereas AUC and Brier score were computed from pooled subject-level out-of-fold predicted probabilities. Lower Brier scores indicate better probabilistic accuracy. Values in parentheses indicate 95% CIs for accuracy, sensitivity, specificity, and AUC. To avoid redundancy, these CIs are reported only for DEP-TFDualNet and the two most competitive deep learning baselines (CNN-LSTM and EEG-Transformer). Single-model CIs for macro-F1 and Brier score are not shown; uncertainty for key between-model differences is summarized in Table 3. The Wilson method was used for accuracy, sensitivity, and specificity, and the DeLong method was used for AUC.

**Table 3:** Subject-level paired bootstrap 95% confidence intervals for between-model performance differences (DEP-TFDualNet minus baseline).

Comparison	Metric	Observed Difference	95% Paired Bootstrap CI
DEP-TFDualNet vs. CNN-LSTM	Accuracy (%)	+8.33	−4.17 to 20.83
DEP-TFDualNet vs. CNN-LSTM	Macro-F1 (%)	+8.43	−3.05 to 19.74
DEP-TFDualNet vs. CNN-LSTM	AUC	+0.05	−0.06 to 0.16
DEP-TFDualNet vs. CNN-LSTM	Brier score	−0.054	−0.103 to −0.008
DEP-TFDualNet vs. EEG-Transformer	Accuracy (%)	+10.42	−2.08 to 22.92
DEP-TFDualNet vs. EEG-Transformer	Macro-F1 (%)	+10.30	−1.18 to 21.68
DEP-TFDualNet vs. EEG-Transformer	AUC	0.00	−0.05 to 0.07
DEP-TFDualNet vs. EEG-Transformer	Brier score	−0.048	−0.093 to 0.004

Note: Observed differences were computed from the original paired subject-level out-of-fold predictions. The corresponding 95% CIs were estimated by subject-level paired bootstrap resampling. Accuracy and macro-F1 are reported as percentage-point differences, whereas AUC and Brier score are reported on the original scale. For Brier score, negative differences favor DEP-TFDualNet because lower values indicate better probabilistic accuracy. Minor discrepancies between the pairwise differences in Table 3 and direct subtraction of the rounded values in Table 2 are due to rounding.

**Table 4:** Results of the component-wise ablation study.

Model	Accuracy (%)	Sensitivity (%)	Specificity (%)	Macro-F1 (%)	AUC	Brier Score
CNN-LSTM	77.08	72.73	80.77	76.83	0.77	0.175
Improved CNN-LSTM	79.17	81.82	76.92	79.13	0.77	0.166
Improved CNN-IndRNN + FC	83.33	81.82	84.62	83.22	0.81	0.141
Improved CNN-IndRNN + KAN	83.33	81.82	84.62	83.22	0.81	0.133
Improved CNN-IndRNN + statistics + FC	85.42	81.82	88.46	85.26	0.81	0.127
DEP-TFDualNet	85.42	81.82	88.46	85.26	0.82	0.121

Note: Improved CNN-LSTM denotes the CNN-LSTM baseline augmented with the proposed front-end enhancement module incorporating multi-scale convolution and dual-domain channel attention. Improved CNN-IndRNN + FC further replaces the LSTM-based temporal modeling module with IndRNNv2. Improved CNN-IndRNN + KAN and Improved CNN-IndRNN + statistics + FC are two parallel ablation variants built on the same Improved CNN-IndRNN backbone, isolating the effects of the KAN-based projection head (followed by linear classification) and the statistical feature branch, respectively. DEP-TFDualNet denotes the full model integrating both enhancements. Accuracy, sensitivity, specificity, and macro-F1 were computed from pooled subject-level out-of-fold predicted labels under five-fold cross-validation, whereas AUC and Brier score were computed from pooled subject-level out-of-fold predicted probabilities. Lower Brier scores indicate better probabilistic accuracy.

**Table 5:** Subject-level paired bootstrap 95% confidence intervals for selected between-model differences in AUC and Brier score in the ablation study.

Comparison	Metric	Observed Difference	95% Paired Bootstrap CI
Improved CNN-IndRNN + KAN vs. Improved CNN-IndRNN + FC	AUC	0.00	−0.06 to 0.07
Improved CNN-IndRNN + KAN vs. Improved CNN-IndRNN + FC	Brier score	−0.008	−0.033 to 0.010
Improved CNN-IndRNN + statistics + FC vs. Improved CNN-IndRNN + FC	AUC	0.00	−0.05 to 0.06
Improved CNN-IndRNN + statistics + FC vs. Improved CNN-IndRNN + FC	Brier score	−0.014	−0.045 to 0.006
DEP-TFDualNet vs. Improved CNN-IndRNN + statistics + FC	AUC	0.01	−0.05 to 0.07
DEP-TFDualNet vs. Improved CNN-IndRNN + statistics + FC	Brier score	−0.006	−0.026 to 0.009

Note: Observed differences were computed from the original paired subject-level out-of-fold predictions. The corresponding 95% CIs were estimated by subject-level paired bootstrap resampling. AUC and Brier score are reported on the original scale. For AUC, positive differences favor the first model in each comparison because higher values indicate better discrimination. For Brier score, negative differences favor the first model because lower values indicate better probabilistic accuracy. Minor discrepancies between the pairwise differences in Table 5 and direct subtraction of the rounded values in Table 4 are due to rounding.

**Table 6:** Leave-one-channel-out analysis of DEP-TFDualNet on the three frontal EEG channels.

Model	Accuracy (%)	Sensitivity (%)	Specificity (%)	Macro-F1 (%)	AUC	Brier Score
DEP-TFDualNet (Fp1 + Fpz + Fp2)	85.42	81.82	88.46	85.26	0.82	0.121
without Fp1	81.25	77.27	84.62	81.04	0.79	0.136
without Fpz	79.17	77.27	80.77	79.02	0.78	0.144
without Fp2	83.33	81.82	84.62	83.22	0.80	0.130

Note: All results were computed from subject-level pooled out-of-fold predictions under the same fixed data partition and evaluation protocol. Accuracy, sensitivity, specificity, and macro-F1 are reported as percentages, whereas AUC and Brier score are reported on the original scale. Higher values indicate better performance for accuracy, sensitivity, specificity, macro-F1, and AUC, whereas lower values indicate better probabilistic accuracy for Brier score.

**Table 7:** Subject-level paired bootstrap 95% confidence intervals for between-model performance differences in the leave-one-channel-out analysis.

Comparison	Metric	Observed Difference	95% Paired Bootstrap CI
DEP-TFDualNet vs. without Fp1	Accuracy (%)	+4.17	−5.25 to 15.58
DEP-TFDualNet vs. without Fp1	AUC	+0.03	−0.03 to 0.14
DEP-TFDualNet vs. without Fp1	Brier score	−0.015	−0.046 to 0.009
DEP-TFDualNet vs. without Fpz	Accuracy (%)	+6.25	−4.17 to 18.75
DEP-TFDualNet vs. without Fpz	AUC	+0.04	−0.04 to 0.13
DEP-TFDualNet vs. without Fpz	Brier score	−0.023	−0.056 to 0.001
DEP-TFDualNet vs. without Fp2	Accuracy (%)	+2.09	−8.33 to 12.50
DEP-TFDualNet vs. without Fp2	AUC	+0.02	−0.06 to 0.09
DEP-TFDualNet vs. without Fp2	Brier score	−0.009	−0.035 to 0.008

Note: Observed differences were computed from the original paired subject-level out-of-fold predictions. The corresponding 95% CIs were estimated by subject-level paired bootstrap resampling. Accuracy is reported as a percentage-point difference, whereas AUC and Brier score are reported on the original scale. For accuracy and AUC, positive differences favor DEP-TFDualNet because higher values indicate better performance. For Brier score, negative differences favor DEP-TFDualNet because lower values indicate better probabilistic accuracy. Minor discrepancies between the pairwise differences in this table and direct subtraction of the rounded values in Table 6 are due to rounding.

**Table 8:** Repeated-run performance of CNN-LSTM and DEP-TFDualNet under fixed subject-level five-fold cross-validation partitions.

Model	Accuracy (%)	Sensitivity (%)	Specificity (%)	Macro-F1 (%)	AUC
CNN-LSTM	76.67 ± 2.85	73.64 ± 4.96	79.62 ± 4.21	76.21 ± 2.97	0.76 ± 0.04
DEP-TFDualNet	84.17 ± 2.14	80.91 ± 3.88	86.92 ± 3.16	83.94 ± 2.26	0.81 ± 0.03

Note: Results are reported as mean ± standard deviation over five repeated runs with different random seeds. The same subject-level five-fold partition was used in all runs. Accuracy, sensitivity, specificity, and macro-F1 were computed from pooled subject-level out-of-fold predicted labels, whereas AUC was computed from pooled subject-level out-of-fold predicted probabilities for the positive class (MDD).

**Table 9:** Quantitative comparison of training accuracy, validation accuracy, and generalization gap between CNN-LSTM and DEP-TFDualNet under the same outer subject-level five-fold cross-validation partition.

Model	Train Accuracy (%)	Validation Accuracy (%)	Generalization Gap (%)
CNN-LSTM	88.1 ± 1.6	76.8 ± 1.8	11.3 ± 1.3
DEP-TFDualNet	93.8 ± 1.4	84.3 ± 1.5	9.5 ± 1.2

Note: Values are reported as mean ± standard deviation across the five outer subject-level cross-validation folds. The generalization gap is defined as training accuracy minus validation accuracy.

**Table 10:** Clinical characteristics of misclassified and correctly classified subjects.

Group	n	PHQ-9	GAD-7	PSQI	CTQ-SF	LES	SSRS	GAD-7/PHQ-9 Ratio
FN	4	9.5 ± 4.7	4.8 ± 4.3	5.0 ± 3.2	42.2 ± 6.5	−13.5 ± 7.9	33.5 ± 11.3	0.58 ± 0.42
FP	3	4.3 ± 1.5	2.3 ± 2.1	4.7 ± 3.2	45.0 ± 17.3	39.0 ± 94.6	42.0 ± 4.4	0.69 ± 0.67
TP	18	19.2 ± 3.2	14.7 ± 4.4	12.7 ± 4.6	52.9 ± 13.3	−61.6 ± 70.7	32.5 ± 7.6	0.76 ± 0.21
TN	23	1.9 ± 1.8	1.5 ± 1.9	3.2 ± 2.1	40.5 ± 5.2	0.7 ± 12.3	41.7 ± 6.6	0.62 ± 0.68

Note: FN, false negative; FP, false positive; TP, true positive; TN, true negative. Data are presented as mean ± standard deviation (SD). PHQ-9, Patient Health Questionnaire-9; GAD-7, Generalized Anxiety Disorder-7; PSQI, Pittsburgh Sleep Quality Index; CTQ-SF, Childhood Trauma Questionnaire-Short Form; LES, Life Events Scale; SSRS, Social Support Rating Scale.

## Data Availability

Publicly available data were analyzed in this study. The MODMA dataset is available at https://modma.lzu.edu.cn/data/index/ (accessed on 10 January 2026).
